# Serial femtosecond crystallography: the first five years

**DOI:** 10.1107/S205225251402702X

**Published:** 2015-02-03

**Authors:** Ilme Schlichting

**Affiliations:** a Department of Biomolecular Mechanisms, Max Planck Institute for Medical Research, Jahnstraße 29, Heidelberg 69120, Germany

**Keywords:** serial femtosecond crystallography, SFX, X-ray lasers, FELs, time-resolved crystallography, microcrystals, radiation damage

## Abstract

Protein crystallography using synchrotron radiation sources has had a tremendous impact on biology, having yielded the structures of thousands of proteins and given detailed insight into their mechanisms. However, the technique is limited by the requirement for macroscopic crystals, which can be difficult to obtain, as well as by the often severe radiation damage caused in diffraction experiments, in particular when using tiny crystals. To slow radiation damage, data collection is typically performed at cryogenic temperatures. With the advent of free-electron lasers (FELs) capable of delivering extremely intense femtosecond X-ray pulses, this situation appears to be remedied, allowing the structure determination of undamaged macromolecules using either macroscopic or microscopic crystals. The latter are exposed to the FEL beam in random orientations and their diffraction data are collected at cryogenic or room temperature in a serial fashion, since each crystal is destroyed upon a single exposure. The new approaches required for crystal growth and delivery, and for diffraction data analysis, including *de novo* phasing, are reviewed. The opportunities and challenges of SFX are described, including applications such as time-resolved measurements and the analysis of radiation damage-prone systems.

## Abstract 2

The advent of hard X-ray free-electron lasers has opened a new chapter in macromolecular crystallography. Recent results, developments and prospects of serial femtosecond crystallography are described.

## 1. Background   

‘It was a wonderful time’, Lawrence Bragg remembered. ‘Like discovering a new goldfield where nuggets could be picked up on the ground, with thrilling new results every week’ (Nobel prize lecture, 1915; http://www.nobelprize.org/nobel_prizes/physics/laureates/1915/wl-bragg-lecture.html). While not as fast-paced as the development of crystallography 100 years ago, a similar spirit of adventure and discovery is found nowadays at X-ray free-electron lasers (XFELs), so-called fourth-generation sources that have become available only recently. XFELs are linear accelerator-based X-ray sources that deliver femtosecond coherent X-ray pulses with a peak brilliance that is nine orders of magnitude higher than that of third-generation synchrotron sources. With these beam characteristics, the two currently available hard X-ray FELs, the Linac Coherent Light Source (LCLS) at SLAC/Stanford, USA (Emma *et al.*, 2010 ▶), and the Spring-8 Ångström Compact Free-electron Laser (SACLA) (Ishikawa *et al.*, 2012 ▶) at Riken/Harima, Japan, allow unprecedented studies in many different areas of science. Targets include atoms, molecules, large non-crystalline particles, liquid, soft and condensed matter, and matter under extreme conditions. Crystalline materials feature prominently too, not only in studies of ultra-fast transitions [see *e.g.* Clark *et al.* (2013 ▶)], but also in the structural analysis of biological molecules (Aquila *et al.*, 2012 ▶; Boutet *et al.*, 2012 ▶; Chapman *et al.*, 2011 ▶; Hirata *et al.*, 2014 ▶; Johansson *et al.*, 2013 ▶; Kern *et al.*, 2012 ▶, 2013 ▶, 2014 ▶; Kupitz, Basu *et al.*, 2014 ▶; Liu, Wacker *et al.*, 2013 ▶; Redecke *et al.*, 2013 ▶; Sawaya *et al.*, 2014 ▶; Suga *et al.*, 2015 ▶; Tenboer *et al.*, 2014 ▶; Weierstall *et al.*, 2014 ▶).

As already pointed out in 1946 by James B. Sumner in his Nobel lecture (http://www.nobelprize.org/nobel_prizes/chemistry/laureates/1946/sumner-lecture.html), the isolation and crystallization of enzymes is challenging because of their often low abundance and stability, making it often difficult if not impossible, even today, to grow large well diffracting crystals of macromolecules. The frequent observation of microcrystalline showers in initial sparse matrix crystallization screens suggests that is should be easier to grow small crystals than larger ones. Thus, it would be very useful to establish diffraction methods that allow the analysis of very small crystals. Moreover, the probability of growth defects should be lower for small crystals than for large ones. Unfortunately, however, radiation damage limits the amount of useful diffraction data that can be obtained from small crystals (Holton & Frankel, 2010 ▶), even when they are kept at cryogenic temperature during data collection to slow the diffusion of radiation-induced radicals.

Solem pointed out in 1986 (Solem, 1986 ▶) that radiation damage can be prevented if the diffraction data are acquired sufficiently rapidly. While this has remained a dream for second- and third-generation synchrotron X-ray sources, it seemed feasible for XFELs in line with molecular dynamics-based simulations performed by Hadju and coworkers (Neutze *et al.*, 2000 ▶). The calculations predicted that useful diffraction data may be obtained from both non-crystalline single particles and nanocrystals, provided the FEL pulse is so brief that it passes through the sample before the onset of significant radiation damage, a concept dubbed diffraction-before-destruction. A proof-of-concept experiment was performed by Chapman and colleagues using FLASH, the soft X-ray FEL in Hamburg, by showing that a nanostructured non-periodic pattern cut in a silicon nitride membrane could be reconstructed by over-sampling techniques using diffraction data acquired with a 32 nm wavelength FEL pulse that destroyed the target upon exposure (Chapman *et al.*, 2006 ▶). The first hard X-ray FEL, the LCLS, came online in 2009, enabling the first experiments with protein crystals (Chapman *et al.*, 2011 ▶). Here, we describe the current state of affairs for macromolecular crystallography at XFELs, summarizing the first five years of this exciting development. Recent reviews include Fromme & Spence (2011 ▶), Patterson (2014 ▶), Schlichting & Miao (2012 ▶) and Spence *et al.* (2012 ▶).

## 2. Samples   

### 2.1. Microcrystals: growth, properties, detection   

**Figure 1:**
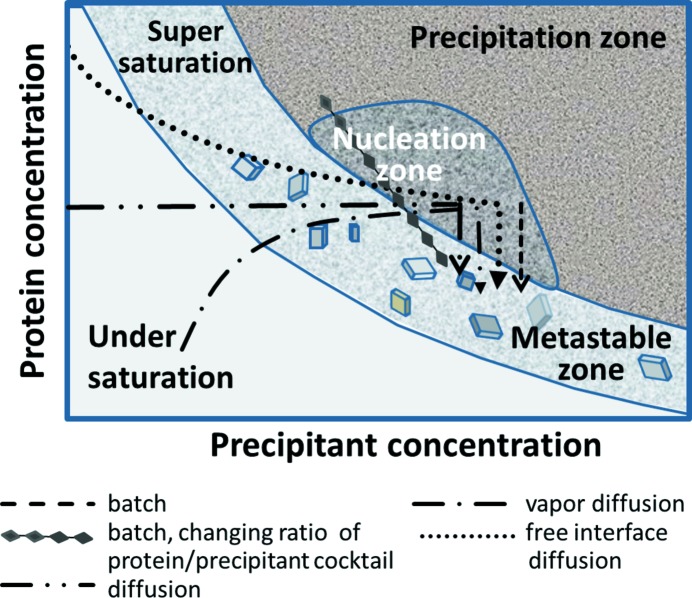
A simplified scheme of a protein crystallization phase diagram. In addition to precipitant concentration, the pH, temperature or additive concentrations are also important adjustable parameters. Crystallization approaches to reach the nucleation and metastable zones are indicated.

From the beginning, crystallographers have aimed to grow large single crystals, with those geared towards neutron crystallography representing the extreme. Accordingly, many tools have been developed over the last few decades to grow macroscopic crystals using less and less material, with typical crystallization drop volumes being 50–200 nl. Microcrystalline showers, frequently observed in initial screening setups, often form the starting point of an optimization protocol to yield large single crystals. However, with the advent of serial femtosecond crystallography (SFX), microcrystals are not only useful by themselves but have become an object of desire and – possibly new – tools are needed to grow them. To this end, and to understand crystallogenesis, it is useful to look at a crystallization phase diagram [see Fig. 1 ▶ and *e.g.* Asherie (2004 ▶)]. Nucleation occurs in a supersaturated phase, while crystal growth takes place in a metastable phase. Thus, for microcrystal growth, a very high degree of supersaturation needs to be established. Since nucleation can be limiting, in particular when it comes to growing a large quantity of microcrystals, it can be extremely useful to use microseeding techniques, which may also increase the likelihood of growing isomorphous microcrystals. Microseeds can be obtained using seed bead kits (Hampton Research) or BeadBug (Benchmark Scientific), depending on the volumes used and number of seeds required.

When starting from a known crystallization condition, it is most convenient to use the batch method for microcrystallization, changing the ratio between the initial protein and precipitant concentrations (see Fig. 1 ▶, diamonds), as well as temperature and pH. The batch method has the advantage that it can be scaled easily from micro- to millilitres. Should it fail, reasonable amounts of microcrystalline material can also be obtained using sitting-drop setups. In both cases, the speed and degree of mixing of the protein and precipitant solutions can have a significant influence on the number of crystals and their size distribution. An alternative approach is crystallization by free-interface diffusion or density centrifugation, described recently in detail for the microcrystallization of photosystem II (Kupitz, Grotjohann *et al.*, 2014 ▶).

Membrane proteins are one of the major targets for SFX structure determination since they often fail to yield large well diffracting crystals. Detergent-solubilized membrane proteins can be crystallized *in surfo* by mixing protein and detergent solutions, using the approaches described above for obtaining microcrystals of soluble proteins. An alternative to using detergents is the stabilization of membrane proteins in bicelles or lipidic mesophases before adding precipitant for *in meso* crystallization. In the case of lipidic cubic phase (LCP) crystallization, setups can be scaled up by injecting the protein-laden LCP as a thin column into a Hamilton syringe filled with precipitant solution (Liu, Wacker *et al.*, 2013 ▶). This not only has the advantage of keeping a rather similar geometry to that used during nanolitre-volume screening setups, but also provides a very convenient means of transferring the microcrystals into the injector sample chamber later on. If injecting the LCP paste into a vacuum for SFX measurements, a thermo­dynamically stable LCP needs to be used (as afforded by 7.9 MAG; Misquitta *et al.*, 2004 ▶) to prevent lamellar phase formation due to evaporative cooling and dehydration (Qiu & Caffrey, 2000 ▶).

The optical identification of microcrystals can be challenging since the crystals often appear to be roundish, similar to a granular precipitate. Particularly difficult is the identification of crystals grown in mesophases such as an LCP. In both cases, imaging using SONICC (second-order nonlinear imaging of chiral crystals) (Wampler *et al.*, 2008 ▶) is very helpful, since this approach can identify crystallinity (depending on the symmetry and orientation of the crystals), while UV light (either directly or obtained by two-photon processes) can be used to distinguish between salt and macromolecules. Submicron particles can be visualized by Nanosight tracking (Malvern Instruments) or dynamic light scattering (DLS) (Kupitz, Grotjohann *et al.*, 2014 ▶) in solution, or by electron microscopy (Stevenson *et al.*, 2014 ▶). This last approach can also be used to check and optimize the quality of nanocrystals (see *e.g.* Cohen *et al.*, 2014 ▶). This can also be done using X-ray powder patterns. While they will not exhibit the same resolution to which single crystals diffract at an FEL, they do show whether the sample is crystalline and can be used to assess the relative diffraction quality of different crystal batches and thus *e.g.* the influence of pH or additives.

In the context of SFX it is of interest to know how common nanocrystals^1^ are compared with larger ones and whether they are better ordered. At present, it is too early to answer this question, but it is difficult to imagine why reasonably ordered nanocrystals would not grow into larger ones, in particular during optimization or other changes in the crystallization solution that change the extent of supersaturation. It has thus been suggested that crystal growths stops due to surface poisoning, resulting in nanocrystals. With decreasing crystal size, the effects of lattice imperfections and disorder of the surface layer become more dominant, affecting the diffraction properties (Dilanian *et al.*, 2013 ▶).

### 2.2. Sample delivery   

**Figure 2:**
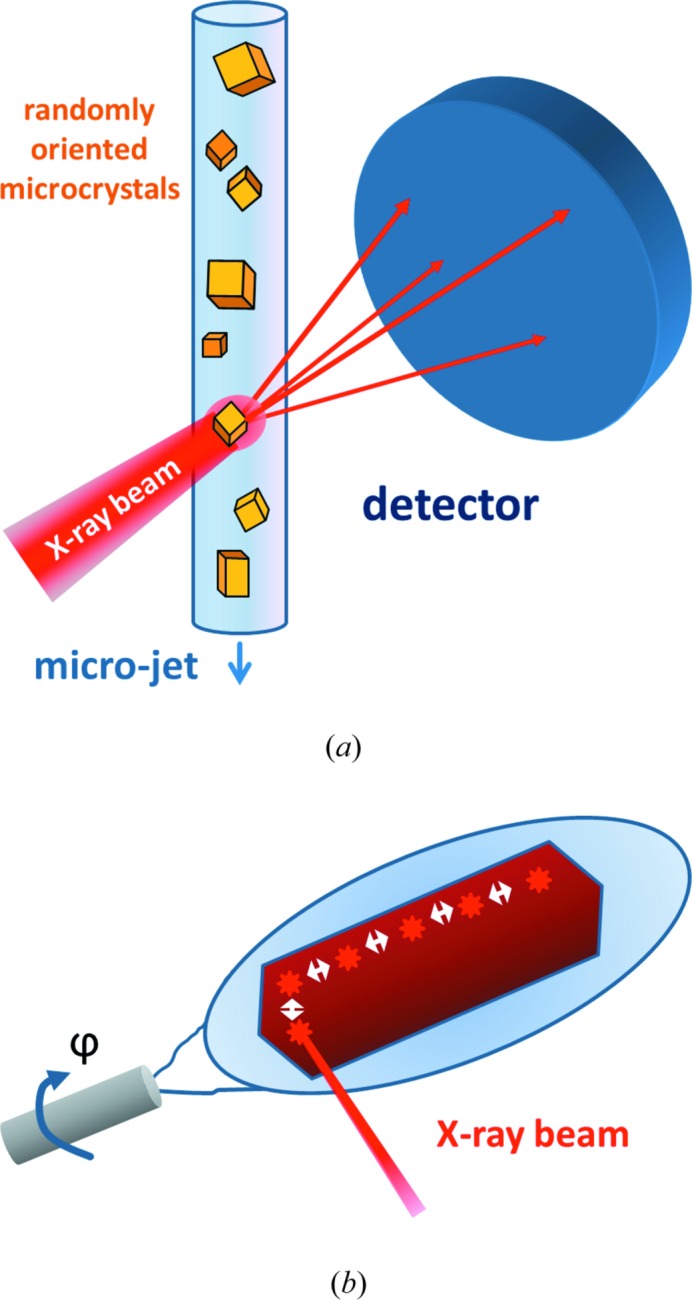
Data collection approaches. (*a*) Randomly oriented micro- and nanocrystals can be delivered into the X-ray interaction region using various forms of liquid jets. (*b*) Large cryo-cooled crystals can be mounted in loops. Serial quasi-rotation data can be collected using a goniometer setup, translating the crystal stepwise by a value Δ*x*,*y*, an experimentally determined damage zone value, while rotating it by Δϕ, a fraction of its experimentally determined mosacity. Other approaches such as chips can also be used.

Macromolecular crystals are characterized by a high solvent content of typically 30–80% and relatively few weak interactions between the molecules forming crystal contacts. On the one hand this is good since it often endows enzyme crystals with catalytic activity, allowing for time-resolved measurements. On the other hand, however, the very nature of a relatively weak lattice renders the crystals sensitive to mechanical stress and changes in the temperature or composition of their mother liquor, in particular dehydration. Thus, sample-delivery techniques need to be gentle, accommodating these variables. Further considerations are the afore­mentioned scarceness of material and the potential requirement to perform such experiments *in vacuo* to reduce background and/or to accommodate detector needs. While conventional goniometer-based capillary or loop mounts can be used for macroscopic crystals (Cohen *et al.*, 2014 ▶; Hirata *et al.*, 2014 ▶; Suga *et al.*, 2015 ▶), new approaches are required when using microcrystals for data collection. A convenient method is sample delivery using liquid jets, since they allow high-throughput replenishment of crystals into the interaction zone (see Fig. 2 ▶ *a*). However, although experimentally straightforward, Rayleigh jets are not suitable for delivering microcrystals of macromolecules. Firstly, they use too much material, and secondly, given the typical composition of protein crystallization cocktails, the probability of ice crystal formation *in vacuo* is rather high. Fortunately, gas dynamic virtual nozzles (GDVN) (DePonte *et al.*, 2008 ▶) provide an elegant solution to both problems. They consist of a relatively large diameter inner capillary for the liquid stream, surrounded by an outer capillary containing helium gas. The helium sheath gas stream is used for focusing the liquid jet which (i) allows the use of relatively large inner diameter capillaries for liquid delivery, reducing the likelihood of clogging while still forming a micron-sized liquid jet; (ii) prevents ice formation *in vacuo*; and (iii) reduces background scattering by producing smaller jets. Settling of the microcrystals in their storage container during data collection – which often takes many hours – can be avoided by using a rotating syringe pump as a sample reservoir (Lomb *et al.*, 2012 ▶). This device can be temperature controlled and addressed by a high-performance liquid chromatography (HPLC) system. GDVN-based microcrystal injection (Weierstall *et al.*, 2012 ▶) has been the most successful and universally applicable method so far, with both soluble and detergent-solubilized membrane protein crystals grown in liquids (Aquila *et al.*, 2012 ▶; Chapman *et al.*, 2011 ▶; Kupitz, Grotjohann *et al.*, 2014 ▶) or in the sponge phase (Johansson *et al.*, 2012 ▶, 2013 ▶) having been injected. However, given that the jet is running continuously at high speed (10 m s^−1^), thus displacing relatively large volumes (10–30 µl min^−1^), and the FEL is pulsed (120 Hz at LCLS, 30 Hz at SACLA), most of the material is wasted, *i.e.* it does not intersect the FEL beam. This may be less of a problem for future FELs with very high repetition rates, such as the European XFEL in Hamburg or LCLSII in Stanford. While rapid sample turnover has advantages when it comes to probing a pristine sample, which is important *e.g.* in pump–probe experiments or for eliminating the effects of radiation damage caused by a previous X-ray exposure, it requires the use of large sample volumes and thus large sample quantities.

The easiest way to reduce sample consumption is to slow the jet. This can be done by using high-viscosity crystal carrier materials, including LCP, either with (Botha *et al.*, 2015 ▶; Weierstall *et al.*, 2014 ▶) or without (Sugahara *et al.*, 2014 ▶) keeping the gas focusing approach. Another low flow rate (0.1–3 µl min^−1^) liquid-jet injection method is based on the principle of electrospinning for focusing the liquid jet. This approach relies on an electric field for focusing and on a high concentration of *e.g.* glycerol, polyethylene glycol or sucrose in the solution containing the crystals, which also helps to prevent them from settling during data collection (Sierra *et al.*, 2012 ▶). An alternative approach to reducing sample consumption by slowing the flow rate is to inject discontinuously, ideally synchronized with the FEL pulses. In this way, the delivery of both sample and X-rays are correlated. One potential way of doing this is by acoustic droplet ejection (Ellson *et al.*, 2003 ▶), with the transducer triggered by the FEL, producing a drop on demand. A possible issue with this approach is the relatively large droplet size (2–10 nl) (Roessler *et al.*, 2013 ▶; Soares *et al.*, 2011 ▶). The droplets may be injected directly into the X-ray beam, similar to the liquid-jet approaches, or deposited on X-ray transparent tape such as Kapton or Mylar, which is then moved into the X-ray beam in a conveyer-belt fashion (Roessler *et al.*, 2013 ▶). The various liquid-jet setups, including droplets, have advantages and disadvantages (Weierstall, 2014 ▶) that include differences in their requirements for the amount of sample and restrictions on the composition of the mother liquor of the crystals. In the absence of flow alignment, they have the advantage of complete sampling of reciprocal space, due to the random orientations of the injected crystals.

Crystals can also be delivered on solid supports (a fixed target or chip). In principle, this allows for a wide range of composition of crystallization solutions and a 100% hit rate if the crystals are located at defined positions. Unless the chips are used at cryogenic temperature, similar to MiTeGen micromesh mounts, they require a means of protecting the crystals from drying. This could be a layer of *e.g.* sugar (Frank *et al.*, 2014 ▶), Paratone-N (Hunter *et al.*, 2014 ▶) or Kapton (Zarrine-Afsar *et al.*, 2012 ▶). Importantly, neither the support substrate nor the shield should add significantly to the X-ray background. Ideally, some kind of self-sorting of the crystals (Zarrine-Afsar *et al.*, 2011 ▶) is used for mounting in the chip or deposition by acoustic droplet ejection (Soares *et al.*, 2011 ▶). Applications of room-temperature measurements using chips at FELs include the analysis of two-dimensional crystals (Frank *et al.*, 2014 ▶), pump–probe measurements, since X-ray exposures of the same crystal can be taken before (attenuated FEL) and after (full FEL beam) laser pumping, and crystal screening. Except for the last case, insufficient sampling of reciprocal space due to crystal alignment for certain crystal morphologies (*e.g.* plates) may be a problem, requiring weakly scattering surface modifications (Zarrine-Afsar *et al.*, 2012 ▶). The effect of shockwaves from the FEL pulses on neighbouring windows needs to be explored (Pardini *et al.*, 2014 ▶).

## 3. Diffraction data   

### 3.1. Diffraction data collection   

XFELs, by their very nature, create femtosecond X-ray pulses of relatively narrow bandwidth. This has implications for the acquisition of diffraction data, since the crystals interact with the X-ray beam only fleetingly. It is thus not possible to rotate crystals during exposure to collect conventional oscillation or rotation data yielding fully integrated reflections. Instead, only partial reflections are obtained. Moreover, the relatively narrow bandwidth reduces the effectiveness of the polychromatic Laue approach in spanning reciprocal space to record fully integrated reflections.

Data-collection strategies depend on whether very small or large crystals are used. Macroscopic crystals can be characterized ahead of XFEL data collection in terms of resolution, mosaicity, orientation *etc.* and this information can be used to devise an optimized crystal-specific quasi-rotation data-collection protocol. For example, to collect high-resolution diffraction data from the highly radiation-sensitive reduced form of bovine cytochrome *c* oxidase (Hirata *et al.*, 2014 ▶) or from the undamaged oxygen-evolving complex of photosystem II (Suga *et al.*, 2015 ▶), large cryo-cooled crystals were mounted on a goniometer and exposed in a serial fashion at SACLA. Consecutive exposures differed by rotational increments of Δϕ and stepwise translations of Δ*x*,*y*, which were guided by experimentally determined mosaicity and damage zone values, respectively (see Fig. 2 ▶ *b*). Thus, in this approach, which can be described as serial femtosecond rotation crystallography (SF-ROX) (Schlichting, 2015 ▶), the orientation of the crystal is known for each individual exposure and conventional processing programs can be used for data analysis. This is not the case when using very small crystals for XFEL data collection or for other schemes that result in the destruction of the crystal upon FEL exposure. In the case of crystals exposed to such a high flux density (irradiance) that they are destroyed upon exposure, replenishment with a fresh crystal in another unknown random orientation is required.

The crystals can be delivered into the FEL beam using liquid microjets, droplets or fixed targets, such as chips (see below) as described in §2.2, and diffraction data are acquired with the speed set by the FEL repetition rate, the detector frame rate or the sample replenishing rate, whatever is rate limiting. The probability of the FEL pulse hitting a crystal in a liquid microjet, droplet or fixed target setup depends on the crystal concentration. Thus, the diffraction patterns collected not only contain the desired single-crystal hits but also empty shots or multiple crystal hits. Therefore, the first step of data analysis consists of identifying crystal diffraction patterns, a process called hit finding. To this end, the distinct sharp features of Bragg peaks are exploited and a threshold (typically 10–30 peaks) is set to identify hits. This data-mining step can be performed offline using the programs *Cheetah* (Barty *et al.*, 2014 ▶) or *CASS* (Foucar *et al.*, 2012 ▶) and the resulting hits can be fed into subsequent analysis programs. *CASS* can also be used for online analysis during data collection. For example, plotting the hit rate helps in optimizing the position of the liquid jet in the FEL beam, displaying the number of saturated pixels helps in adjusting the flux by correct choice of attenuators, and summing up Bragg peaks in the form of virtual powder patterns provides a fast means of judging data completeness.

In general, SFX data sets consist of a large number of independent snapshot or still diffraction patterns, each collected from a randomly oriented crystal that is effectively standing still during the femtosecond X-ray exposure which ultimately destroys it. The crystals differ not only in their orientation but also in their size and quality. This affects the recorded diffraction intensities, which also depend on whether the crystal is located at the periphery of the X-ray beam or its centre and on whether the energy of the X-ray pulse is high or low. All of these, as well as the changing spectral distribution of the self-amplified spontaneous emission (SASE) FEL beam, result in a great deal of fluctuation in the recorded intensities. Indexing of SFX data can also be challenging, in particular in the case of small unit cells, due to the possibly significantly reduced number of Bragg reflections compared with rotation data. A further complication occurs in the indexing of diffraction data from crystals where the symmetry of the Bravais lattice is higher than that of the space group. This results in an indexing ambiguity for 27 space groups [see Brehm & Diederichs (2014 ▶) and White *et al.* (2013 ▶) for a list]. A decision between possible indexing modes has to be made for each data set that is to be merged or compared with other data sets, which is not a problem for conventional (partial) data sets. In the case of SFX data, this decision has to be made for each diffraction pattern, and failure results in data sets that appear to be perfectly twinned (50%). For all of these reasons, SFX data reduction requires specialized analysis programs.

### 3.2. Analysis of SFX data   

Currently, three different programs [*CrystFEL* (White *et al.*, 2012 ▶; White *et al.*, 2013 ▶), *cctbx.xfel* (Hattne *et al.*, 2014 ▶; Sauter *et al.*, 2013 ▶) and *nXDS* (Kabsch, 2014 ▶)] are available for the analysis of serial diffraction data and they differ in their approaches and algorithms. *CrystFEL*, the first one available, relies on other programs [*e.g.* *Cheetah* (Barty *et al.*, 2014 ▶) or *CASS* (Foucar *et al.*, 2012 ▶)] for hit identification. *CrystFEL* identifies Bragg peaks in the hits, and passes lists of reflections to *MOSFLM* (Powell *et al.*, 2013 ▶) or *DIRAX* (Duisenberg, 1992 ▶) for indexing and integration and merges the partial diffraction intensities in a Monte Carlo-like fashion (Kirian *et al.*, 2010 ▶, 2011 ▶). A common resolution limit is determined for all diffraction patterns included in the data set. This approach can result in relatively high Wilson *B* factors, a relatively low signal-to-noise ratio, very high completeness and multiplicity of observations in the high-resolution shells, due to the possible inclusion of weak reflections or even noise from patterns with either poorly or weakly diffracting crystals. In contrast, both *nXDS* and *cctbx.xfel* determine the resolution cut-off for each diffraction pattern individually, which results in lower multiplicities and higher signal-to-noise ratios of reflections in the high-resolution range and lower Wilson *B* factors compared with *CrystFEL*. While *nXDS* uses global scaling, profile fitting and post-refinement, neither *CrystFEL* nor *cctbx.xfel* does so yet. A systematic comparison of SFX data analysed using both *CrystFEL* and *cctbx.xfel* has been published recently, pointing out these and other differences (Sawaya *et al.*, 2014 ▶). *nXDS* builds on *XDS* (Kabsch, 1988 ▶), but it has a number of new features that deal explicitly with the properties of SFX data mentioned above. In particular, *nXDS* does not rely on the Monte Carlo method for integration. After indexing, the pixel content of a reflection is mapped to the Ewald sphere, followed by profile fitting assuming a Gaussian rocking curve. Instead of using the concept of partiality, an Ewald offset correction factor is introduced that estimates the angular distance of the reflection from the Ewald sphere. This allows post-refinement of all diffraction and scaling parameters of the globally scaled raw intensities to derive structure factor amplitudes.

Three approaches have been developed to resolve the indexing ambiguity of diffraction patterns in the case of crystals that have a lower point group than the lattice symmetry. *nXDS* uses either correlations with a reference data set or, in its absence, a selective breeding algorithm. Brehm & Diederichs (2014 ▶) used pairwise correlations of patterns and clustering of patterns that have been indexed in the same way. This approach has been implemented in *CrystFEL* and *cctbx.xfel*. Liu & Spence (2014 ▶) proposed an algorithm based on an expectation maximization algorithm, which has been tested on simulated data.

### 3.3. Quality of SFX data   

Given the many fluctuations inherent in SFX, data quality is of particular interest. This was analysed using the well established lysozyme model system that yields both well diffracting macroscopic and microscopic crystals. A comparison of high-resolution SFX and conventional rotation data sets collected at the LCLS and Swiss Light Source, respectively, showed good agreement of both the diffraction intensities and their statistics, as well as of the refined models (Boutet *et al.*, 2012 ▶). The synchrotron measurements were performed using a single large crystal kept at room temperature, resulting in a total dose of 24 kGy, while the SFX measurements were performed using a microcrystalline slurry of lysozyme microcrystals that was injected into the FEL beam using a liquid jet. Some 20 000 individual diffraction patterns were indexed and scaled, each originating from a single randomly oriented crystal that experienced a dose of 33 or 2.9 MGy for a FEL pulse length of 40 or 5 fs, respectively. Importantly, the difference electron density maps using synchrotron and FEL data [*F*_obs_(SLS) − *F*_obs_(LCLS)] showed no sign of radiation damage. The structure of hen egg-white lysozyme (HEWL) was solved by molecular replacement using a turkey lysozyme model, and there was a clear difference in electron density in the case of side-chain variations. Also, the sulfur atoms in the S bridges were distinct, in line with a resolution of 1.9 Å (Boutet *et al.*, 2012 ▶).

While SFX data are good enough to allow observation of new structural features that were not part of the model (Redecke *et al.*, 2013 ▶), as well as anomalous differences from both sulfur (Barends *et al.*, 2013 ▶) and metals (Barends *et al.*, 2014 ▶; Kern *et al.*, 2014 ▶), they do not seem to reach the quality of synchrotron data. Given all the fluctuations that are part of the experiment [X-rays (photon energy and spectral distribution, pulse energy), crystals (size, quality) and even the detector (gain, metrology)], this can have many causes, most of which are not well characterized. Therefore, in a well defined control experiment aimed at testing the influence of merging a very large number of still diffraction patterns, Kabsch (2014 ▶) analysed a fine-sliced (Δϕ = 0.02°) synchrotron data set of a selenomethione-labelled protein crystal consisting of 20 000 consecutive rotation images using *XDS* (consecutive images) and *nXDS* (randomized images). These showed good agreement, with correct indexing despite an indexing ambiguity (the crystal has *P*4_3_ space-group symmetry, which is lower than the 422 lattice symmetry, implying a twofold indexing ambiguity) and a strong anomalous signal that allowed *de novo* phasing in both cases. Nevertheless, *nXDS* resulted in an almost threefold reduced mean signal-to-noise ratio than *XDS*. The lower accuracy of *nXDS* presumably results from two-dimensional instead of three-dimensional profile fitting and a lack of other not yet implemented corrections.

Intuitively, one would expect a large influence of the changing spectral distribution of the X-ray pulses, but this does not seem to be the case when comparing SFX data from lysozyme microcrystals collected with a SASE (fluctuating polychromatic distribution) or seeded (more or less monochromatic) beam (Amann *et al.*, 2012 ▶) and analysed with *CrystFEL* (Barends *et al.*, 2015 ▶). This result points to other sources of error in the data. Merging data from several crystals is a common approach in conventional macromolecular crystallography to alleviate the effects of radiation damage. Great care is taken as to which partial data sets are merged to account for *e.g.* non-isomorphism (Foadi *et al.*, 2013 ▶), in particular for single-wavelength anomalous diffraction (SAD) phasing (Liu *et al.*, 2012 ▶; Liu, Liu & Hendrickson, 2013 ▶). While this is so far not the case for SFX data using the Monte Carlo approach, no effect on data quality was observed when classifying lysozyme SFX data into different groups of unit-cell distributions (Barends *et al.*, 2015 ▶).

### 3.4. Phasing   

Crystallographic structure determination requires the retrieval of phases which are lost during the measurement of diffraction intensities. Conventional phasing methods rely on contributions from heavy-atom scatterers, such as multiple or single isomorphous replacement approaches, and/or on multi- or single-wavelength anomalous diffraction (MAD or SAD) measurements, which exploit element-specific scattering from X-ray absorption edges. There is no reason why these approaches should not work with SFX data. Indeed, it was demonstrated recently that not only can the very weak anomalous diffraction from endogenous sulfur atoms in a protein be measured (Barends *et al.*, 2013 ▶) but also that SFX data are accurate enough for SAD phasing (Barends *et al.*, 2014 ▶). To this end, a high-multiplicity 2.1 Å resolution SFX data set of a lysozyme heavy-atom derivative was collected that gives a strong anomalous signal from two gadolinium atoms per asymmetric unit (Girard *et al.*, 2003 ▶). As expected for SFX data (Kirian *et al.*, 2010 ▶, 2011 ▶; White *et al.*, 2012 ▶), the data quality depended strongly on the number of integrated patterns. While the two gadolinium atoms could be found easily in phased maps using a couple of thousand indexed patterns, solving of the substructure and *de novo* phasing were only possible when all ∼60 000 patterns were used, yielding excellent values of *R*_split_ as well as a high anomalous correlation coefficient CC_ano_ of 0.48 (Barends *et al.*, 2014 ▶).

While the gadolinium derivative allowed *de novo* phasing, it was surprisingly difficult given the strong anomalous signal of the two gadolinium atoms. There are several reasons why phasing of SFX data using anomalous differences can be challenging, including the small magnitude of the signal, possible merging of data from crystals that are non-isomorphous, incorrect measurements of Friedel pairs in the case of a low multiplicity of measurements which will result in artificially large anomalous differences and, importantly, a lack of a good measure for the errors [σ(*I*)] of the intensities, in particular when using Monte Carlo approaches for integration. However, SAD approaches rely on a good error model of the measured amplitudes (McCoy, 2004 ▶; McCoy *et al.*, 2004 ▶).

New phasing approaches have been proposed for SFX data that make explicit use of the unique properties of FEL radiation. The coherent nature of the FEL beam allows measurement of the inter-Bragg intensities in diffraction patterns from nanocrystals (Chapman *et al.*, 2011 ▶), caused by the convolution of the crystal-shape transform function of a finite crystal with the Bragg peak, modulated by the molecular transform. While the effects of incomplete unit cells (Kirian *et al.*, 2014 ▶) or surface layer contributions (Dilanian *et al.*, 2013 ▶) complicate the situation, this approach allows phasing by oversampling techniques, exploiting the shape transform function derived from the diffraction patterns (Spence *et al.*, 2011 ▶) or the gradients of the diffraction intensities at the Bragg position, resulting in small shifts of the peaks (Elser, 2013 ▶), as reviewed and discussed by Millane & Chen (2014 ▶). The high peak brilliance of the FEL can be used for variations of radiation-induced phasing (Banumathi *et al.*, 2004 ▶; Ravelli *et al.*, 2003 ▶), exploiting the high fluence of FELs specifically to introduce significant electronic damage by multiple ionizations of heavy atoms for photon energies close to inner-shell absorption edges. The scattering cross sections of a heavy atom below and above the *K* edge change in a wavelength- and intensity-dependent manner which can be used for a high-intensity MAD phasing approach using modified Karle–Hendrickson equations (Son *et al.*, 2011 ▶). The calculations assume electronic changes only for the absorbing heavy atom, affecting its anomalous scattering behaviour, but this may not be the case. In particular, the effects of Bragg termination may change the intensity distribution between high- and low-resolution reflections (Barty *et al.*, 2012 ▶; Lomb *et al.*, 2011 ▶) and thereby introduce an apparent non-isomorphism.

### 3.5. Radiation damage   

Radiation damage has plagued macromolecular crystallography since the very beginning (Blake & Phillips, 1962 ▶). It is particularly problematic for the study of redox-sensitive systems such as metalloproteins – with photosystem II (PSII; Yano *et al.*, 2005 ▶) being the poster child – and for nano- to micron-sized crystals in general (Holton & Frankel, 2010 ▶). X-ray FELs, due to their high peak brilliance and femtosecond pulses, have been presented as a solution to this problem, allowing the diffraction-before-destruction approach that outruns radiation damage (Neutze *et al.*, 2000 ▶). While this seems to be true for ‘moderate’ doses of 30–150 MGy per crystal exposure (Boutet *et al.*, 2012 ▶; Kern *et al.*, 2013 ▶), it does not seem to be the case for higher doses (Barty *et al.*, 2012 ▶; Lomb *et al.*, 2011 ▶). Both dose- and dose-rate dependent resolution deterioration of the diffraction data of lysozyme microcrystals (Lomb *et al.*, 2011 ▶) and photosystem I (PSI) (Barty *et al.*, 2012 ▶) were observed for doses of several GGy. The two studies agreed on the underlying reason for this observation, namely the resolution-dependent disordering of the crystal lattice with exposure time. However, they arrived at different conclusions. Assuming a homogeneous distribution of atoms/elements in the unit cell, Barty *et al.* (2012 ▶) concluded that FEL-induced disorder gates the diffraction, with undamaged high-resolution intensities collected at the beginning of the pulse being superimposed on increasingly damaged lower-resolution intensities towards the end of the pulse and correctable by Wilson-type scaling. While Lomb *et al.* (2011 ▶) agreed with this underlying reason for global damage, they predicted the existence of local damage hotspots which deteriorate the data in a manner that cannot be rectified. Since the resolution of the data prevented further analysis, this issue is currently being explored.

Safe dose limits were established experimentally for synchrotron data collection at cryogenic temperatures (30 Mgy) (Owen *et al.*, 2006 ▶) and room temperature (0.2 MGy) (Owen *et al.*, 2012 ▶), but such experimentally determined numbers are missing for the femtosecond exposures and dose rates provided by FELs. Based on calculations, Chapman and coworkers deduced that, for a typical protein crystal, each atom is ionized once at the end of the FEL pulse at a dose of 400 MGy (Chapman *et al.*, 2014 ▶). Thus, during the pulse a photon is more likely to be scattered from a neutral atom than an ionized atom. At higher doses, it is expected that the pulse-integrated diffraction will be affected by the ionizations.

## 4. Applications   

SFX allows structural analysis beyond the limitations conventionally set by radiation damage. This includes the photoreduction of redox-active systems such as metallo­proteins. For example, there has been controversy over the nature of the ligand in bovine cytochrome *c* oxidase. Recent high-resolution SF-ROX measurements at SACLA using large cryo-cooled crystals are in line with a peroxide ligand in the fully oxidized resting state, instead of a – presumably radiation-induced – hydroxide ligand (Hirata *et al.*, 2014 ▶). Using large (cryo-cooled) crystals for SFX measurements has the advantage that conventional crystal-optimization procedures such as dehydration can be used, which can result in a significant increase in resolution, with PSII being a prominent example (Umena *et al.*, 2011 ▶). Using the SF-ROX approach and large dehydrated PSII crystals resulted in a high-resolution structure of the undamaged oxygen-evolving complex. Interestingly, the manganese–manganese distances are somewhat shorter in the undamaged structure and one of the oxo-bridged O atoms (O5) has unusually long distances to the manganese ions, suggesting that it is a hydroxide ion instead of an oxygen dianion. This would imply that O5 is possibly one of the substrate oxygen atoms (Suga *et al.*, 2015 ▶). That FEL measurements can indeed provide damage-free data for the oxygen-evolving complex of PSII has been shown by X-ray emission spectroscopy performed in parallel with SFX measurements on PSII microcrystals at LCLS (50 fs X-ray pulses of 7 keV photon energy, dose up to 150 MGy per crystal) (Kern *et al.*, 2013 ▶), which is in stark contrast with measurements performed at synchrotrons using cryo-cooled macroscopic PSII crystals (Yano *et al.*, 2005 ▶). While the analysis of microcrystals has the advantage of allowing efficient optical excitation for time-resolved measurements, studies using PSII microcrystals are currently hampered by low (4.5 to 5 Å) resolution (Kern *et al.*, 2014 ▶; Kupitz, Basu *et al.*, 2014 ▶).

This is in line with anecdotal evidence from recent years, which indicates that: (i) small microcrystals may not diffract to as high a resolution as carefully optimized macroscopic crystals; (ii) there is a correlation between crystal size and resolution for systems that crystallize (relatively) easily, for example our microcrystals of myoglobin and ferredoxin only diffract to high resolution when thicker than 2 µm (unpublished results); and (iii) crystals which are restricted in their size by external parameters, such as crystals grown *in vivo* or in an LCP, often yield better data at FELs than at synchrotrons. This seems to suggest that in case (iii), but not (ii), it is radiation damage that limits the resolution attainable using synchrotron sources, not crystal order. In conclusion, crystal quality also remains a limiting factor for SFX.

The ultra-short duration of FEL pulses extends the ∼100 ps time resolution afforded by synchrotron-based Laue experiments – which is limited by the electron bunch length – to the chemical timescale of femtoseconds. This allows researchers to follow *e.g.* the very early events upon breaking of the haem iron–carbon monoxide bond in carbonmonoxy myoglobin (Schotte *et al.*, 2003 ▶), or the isomerization of the cofactor in photoactive yellow protein (Jung *et al.*, 2013 ▶; Schotte *et al.*, 2012 ▶), as demonstrated recently in a proof-of-principle experiment at the LCLS (Tenboer *et al.*, 2014 ▶). Moreover, since SFX relies anyway on replenishing crystals in a high-throughput fashion by a rapidly flowing jet, the method is perfectly suited to the study of irreversible reactions, *e.g.* triggered by photolysis of a cage compound (Schlichting *et al.*, 1990 ▶), including those that result in destruction of the crystal (Aquila *et al.*, 2012 ▶). The possibility of analysing very small crystals alleviates some of the issues connected with the study of macroscopic crystals, such as high optical density limiting the extent of excitation in pump–probe experiments, and diffusion times for soaking experiments limiting the reaction timescales that can be followed.

### 4.1. Cross-fertilization of techniques   

It is interesting to see that SFX data-collection approaches developed for FELs out of necessity are now being adapted for serial data collection at synchrotron sources. The motivation is similar, a high-throughput high dose-rate approach for data collection from small or weakly scattering crystals using the unattenuated X-ray beam. The full tolerable dose can be used for each single-crystal exposure, given the continuous supply of fresh crystals. To this end, different approaches have been developed: (i) cryo-cooled crystalline slurries suspended in loops (Gati *et al.*, 2014 ▶); (ii) slurries of crystals in capillaries (Stellato *et al.*, 2014 ▶); (iii) chip mounts (Zarrine-Afsar *et al.*, 2012 ▶); and (iv) high-viscosity extrusion injectors (Botha *et al.*, 2015 ▶). In the first three cases, the sample holder (loop, capillary, chip) is moved sequentially through the X-ray beam, while it is the high-viscosity carrier medium which moves in case (iv). A high-viscosity medium such as an LCP or grease (Sugahara *et al.*, 2014 ▶) accommodates the greatly protracted exposure time (milliseconds at synchrotrons *versus* femto­seconds at FELs) by passing the crystals slowly through the X-ray beam, while avoiding unwanted rotation that would smear out the diffraction peaks. This approach has the advantage of exposing a pristine sample, which is not only important for reducing damage effects but also for time-resolved pump–probe measurements. First results obtained at the Swiss Light Source using this high-viscosity extrusion injection-based serial crystallography (SX) approach, including a demonstration of *de novo* phasing, have been described by Botha *et al.* (2015 ▶). While all SX approaches require further development to reduce background scattering, they represent an interesting alternative to FEL data collection to investigate the structure and dynamics of macromolecules at ambient temperature, in particular in view of the ongoing developments in detectors and synchrotron sources (*e.g.* diffraction-limited storage rings and ‘pink’ beams).

In conclusion, the future of macromolecular crystallography is very bright.

## Footnotes

1 The term ‘nanocrystals’ is ill-defined in the SFX community. Often, it is used for crystals used in SFX experiments, independent of whether they are submicron or micron-sized. This is also reflected in the fact that SFX is often called femtosecond nanocrystallography (Fromme & Spence, 2011 ▶). In this review, nanocrystals are defined as submicron-sized crystals.

## References

[bb1] Amann, J. *et al.* (2012). *Nat. Photonics*, **6**, 693–698.

[bb2] Aquila, A. *et al.* (2012). *Opt. Express*, **20**, 2706–2716. 10.1364/OE.20.002706 PMC3413412 22330507

[bb98] Asherie, N. (2004). *Methods*, **34**, 266–272. 10.1016/j.ymeth.2004.03.028 15325646

[bb3] Banumathi, S., Zwart, P. H., Ramagopal, U. A., Dauter, M., & Dauter, Z. (2004). *Acta Cryst.* D**60**, 1085–1093. 10.1107/S0907444904007917 15159568

[bb4] Barends, T. R., Foucar, L., Botha, S., Doak, R. B., Shoeman, R. L., Nass, K., Koglin, J. E., Williams, G. J., Boutet, S., Messerschmidt, M., & Schlichting, I. (2014). *Nature*, **505**, 244–247. 10.1038/nature12773 24270807

[bb5] Barends, T. R. *et al.* (2013). *Acta Cryst.* D**69**, 838–842.

[bb6] Barends, T. R. *et al.* (2015). *J. Synchrotron Rad.* Submitted.

[bb7] Barty, A. *et al.* (2012). *Nat. Photonics*, **6**, 35–40. 10.1038/nphoton.2011.297 PMC3783007 24078834

[bb8] Barty, A., Kirian, R. A., Maia, F. R. N. C., Hantke, M., Yoon, C. H., White, T. A., & Chapman, H. (2014). *J. Appl. Cryst.* **47**, 1118–1131. 10.1107/S1600576714007626 PMC4038800 24904246

[bb9] Blake, C. C. F. & Phillips, D. C. (1962). *Effects of X-irradiation on Single Crystals of Myoglobin* *Biological Effects of Ionizing Radiation at the Molecular Level*, pp. 183–191. Vienna: International Atomic Energy Agency.

[bb10] Botha, S., Nass, K., Barends, T. R., Kabsch, W., Latz, B., Dworkowksi, F., Foucar, L., Panepucci, E. H., Wang, M., Shoeman, R. L., Schlichting, I. & Doak, R. B. (2015). *Acta Cryst.* D**71**, 387–397. 10.1107/S1399004714026327 25664750

[bb11] Boutet, S. *et al.* (2012). *Science*, **337**, 362–364.

[bb12] Brehm, W. & Diederichs, K. (2014). *Acta Cryst.* D**70**, 101–109. 10.1107/S1399004713025431 24419383

[bb13] Chapman, H. N. *et al.* (2006). *Nat. Phys.* **2**, 839–843.

[bb14] Chapman, H. N., Caleman, C. & Timneanu, N. (2014). *Philos. Trans. R. Soc. London Ser. B*, **369**, 20130313. 10.1098/rstb.2013.0313 PMC4052855 24914146

[bb15] Chapman, H. N. *et al.* (2011). *Nature*, **470**, 73–77. 10.1038/nature09750 PMC3429598 21293373

[bb16] Clark, J. N., Beitra, L., Xiong, G., Higginbotham, A., Fritz, D. M., Lemke, H. T., Zhu, D., Chollet, M., Williams, G. J., Messerschmidt, M., Abbey, B., Harder, R. J., Korsunsky, A. M., Wark, J. S. & Robinson, I. K. (2013). *Science*, **341**, 56–59. 10.1126/science.1236034 23704372

[bb17] Cohen, A. E. *et al.* (2015). *Proc. Natl. Acad. Sci USA*, **111**, 17122–17127.

[bb18] DePonte, D. P., Weierstall, U., Schmidt, K., Warner, J., Starodub, D., Spence, J. C. H. & Doak, R. B. (2008). *J. Phys. D Appl. Phys.* **41**, 195505.

[bb19] Dilanian, R. A., Streltsov, V. A., Quiney, H. M. & Nugent, K. A. (2013). *Acta Cryst.* A**69**, 108–118. 10.1107/S0108767312042535 23250067

[bb20] Duisenberg, A. J. M. (1992). *J. Appl. Cryst.* **25**, 92–96.

[bb21] Ellson, R., Mutz, M., Browning, B., Lee, L. Jr, Miller, M. F. & Papen, R. (2003). *J. Lab. Autom.* ***8***, 29–34.

[bb22] Elser, V. (2013). *Acta Cryst.* A**69**, 559–569. 10.1107/S0108767313023362 24132217

[bb23] Emma, P. *et al.* (2010). *Nat. Photonics*, **4**, 641–647.

[bb24] Foadi, J., Aller, P., Alguel, Y., Cameron, A., Axford, D., Owen, R. L., Armour, W., Waterman, D. G., Iwata, S. & Evans, G. (2013). *Acta Cryst.* D**69**, 1617–1632. 10.1107/S0907444913012274 PMC3727331 23897484

[bb25] Foucar, L., Barty, A., Coppola, N., Hartmann, R., Holl, P., Hoppe, U., Kassemeyer, S., Kimmel, N., Kupper, J., Scholz, M., Techert, S., White, T. A., Struder, L. & Ullrich, J. (2012). *Comput. Phys. Commun.* **183**, 2207.

[bb26] Frank, M. *et al.* (2014). *IUCrJ*, **1**, 95–100. 10.1107/S2052252514001444 PMC4062087 25075325

[bb27] Fromme, P. & Spence, J. C. (2011). *Curr. Opin. Struct. Biol.* **21**, 509–516. 10.1016/j.sbi.2011.06.001 PMC3413407 21752635

[bb28] Gati, C., Bourenkov, G., Klinge, M., Rehders, D., Stellato, F., Oberthur, D., Yefanov, O., Sommer, B. P., Mogk, S., Duszenko, M., Betzel, C., Schneider, T. R., Chapman, H. N. & Redecke, L. (2014). *IUCrJ*, **1**, 87–94. 10.1107/S2052252513033939 PMC4062088 25075324

[bb29] Girard, E., Stelter, M., Anelli, P. L., Vicat, J. & Kahn, R. (2003). *Acta Cryst.* D**59**, 118–126. 10.1107/s0907444902020176 12499547

[bb30] Hattne, J. *et al.* (2014). *Nat. Methods*, **11**, 545–548. 10.1038/nmeth.2887 PMC4008696 24633409

[bb31] Hirata, K. *et al.* (2014). *Nat. Methods*, **11**, 734–736. 10.1038/nmeth.2962 24813624

[bb32] Holton, J. M. & Frankel, K. A. (2010). *Acta Cryst.* D**66**, 393–408. 10.1107/S0907444910007262 PMC2852304 20382993

[bb33] Hunter, M. S. *et al.* (2014). *Sci. Rep.* **4**, 6026. 10.1038/srep06026 PMC4129423 25113598

[bb34] Ishikawa, T. *et al.* (2012). *Nat. Photonics*, **6**, 540–544.

[bb35] Johansson, L. C. *et al.* (2013). *Nat. Commun.* **4**, 2911. 10.1038/ncomms3911 PMC3905732 24352554

[bb36] Johansson, L. C. *et al.* (2012). *Nat. Methods*, **9**, 263–265. 10.1038/nmeth.1867 PMC3438231 22286383

[bb37] Jung, Y. O., Lee, J. H., Kim, J., Schmidt, M., Moffat, K., Srajer, V. & Ihee, H. (2013). *Nat. Chem.* **5**, 212–220. 10.1038/nchem.1565 PMC3579544 23422563

[bb38] Kabsch, W. (1988). *J. Appl. Cryst.* **21**, 916–924.

[bb39] Kabsch, W. (2014). *Acta Cryst.* D**70**, 2204–2216. 10.1107/S1399004714013534 PMC4118830 25084339

[bb41] Kern, J. *et al.* (2012). *Proc. Natl. Acad. Sci. USA*, **109**, 9721–9726.

[bb40] Kern, J. *et al.* (2013). *Science*, **340**, 491–495.

[bb43] Kern, J. *et al.* (2014). *Nat. Commun.* **5**, 4371. 10.1038/ncomms5371 PMC4151126 25006873

[bb44] Kirian, R. A., Bean, R. J., Beyerlein, K. R., Yefanov, O. M., White, T. A., Barty, A. & Chapman, H. N. (2014). *Philos. Trans. R. Soc. London Ser. B*, **369**, 20130331. 10.1098/rstb.2013.0331 PMC4052867 24914158

[bb45] Kirian, R. A., Wang, X., Weierstall, U., Schmidt, K. E., Spence, J., Hunter, M., Fromme, P., White, T. A., Chapman, H. & Holton, J. M. (2010). *Opt. Express*, **18**, 5713–5723. 10.1364/OE.18.005713 PMC4038330 20389587

[bb46] Kirian, R. A. *et al.* (2011). *Acta Cryst.* A**67**, 131–140. 10.1107/S0108767310050981 PMC3066792 21325716

[bb47] Kupitz, C., Basu, S. *et al.* (2014). *Nature*, **513**, 261–265. 10.1038/nature13453 PMC4821544 25043005

[bb48] Kupitz, C., Grotjohann, I., Conrad, C. E., Roy-Chowdhury, S., Fromme, R. & Fromme, P. (2014). *Philos. Trans. R. Soc. London Ser. B*, **369**, 20130316. 10.1098/rstb.2013.0316 PMC4052858 24914149

[bb49] Liu, Q., Dahmane, T., Zhang, Z., Assur, Z., Brasch, J., Shapiro, L., Mancia, F. & Hendrickson, W. A. (2012). *Science*, **336**, 1033–1037. 10.1126/science.1218753 PMC3769101 22628655

[bb50] Liu, Q., Liu, Q. & Hendrickson, W. A. (2013). *Acta Cryst.* D**69**, 1314–1332. 10.1107/S0907444913001479 PMC3689535 23793158

[bb51] Liu, H. & Spence, J. H. C. (2014). *IUCrJ*, **1**, 393–401. 10.1107/S2052252514020314 PMC4224458 25485120

[bb52] Liu, W., Wacker, D. *et al.* (2013). *Science*, **342**, 1521–1524. 10.1126/science.1244142 PMC3902108 24357322

[bb53] Lomb, L. *et al.* (2011). *Phys. Rev. B*, **82**, 214111.

[bb54] Lomb, L., Steinbrener, J., Beisel, D., Berndt, D., Kieser, C., Lukat, M., Neef, N. & Shoeman, R. L. (2012). *J. Appl. Cryst.* **45**, 674–678.

[bb55] McCoy, A. J. (2004). *Acta Cryst.* D**60**, 2169–2183. 10.1107/S0907444904016038 15572770

[bb56] McCoy, A. J., Storoni, L. C. & Read, R. J. (2004). *Acta Cryst.* D**60**, 1220–1228. 10.1107/S0907444904009990 15213383

[bb57] Millane, R. P. & Chen, J. P. L. (2014). *Philos. Trans. R. Soc. London Ser. B*, **369**, 20130498. 10.1098/rstb.2013.0498 PMC4052874 24914165

[bb58] Misquitta, Y., Cherezov, V., Havas, F., Patterson, S., Mohan, J. M., Wells, A. J., Hart, D. J. & Caffrey, M. (2004). *J. Struct. Biol.* **148**, 169–175. 10.1016/j.jsb.2004.06.008 15477097

[bb59] Neutze, R., Wouts, R., van der Spoel, D., Weckert, E. & Hajdu, J. (2000). *Nature*, **406**, 752–757. 10.1038/35021099 10963603

[bb61] Owen, R. L., Axford, D., Nettleship, J. E., Owens, R. J., Robinson, J. I., Morgan, A. W., Dore, A. S., Lebon, G., Tate, C. G., Fry, E. E., Ren, J., Stuart, D. I., & Evans, G. (2012). *Acta Cryst.* D**68**, 810–818. 10.1107/S0907444912012553 PMC4791751 22751666

[bb62] Owen, R. L., Rudino-Pinera, E. & Garman, E. F. (2006). *Proc. Natl. Acad. Sci. USA*, **103**, 4912–4917. 10.1073/pnas.0600973103 PMC1458769 16549763

[bb63] Pardini, T., Boutet, S., Bradley, J. A., Doeppner, T., Fletcher, L. B., Gardner, D. F., Hill, R. M., Hunter, M. S., Krzywinski, J., Messerschmidt, M., Pak, A. E., Quirin, F., Sokolowski-Tinten, K., Williams, G. & Hau-Riege, S. P. (2014). *Phys. Rev. Appl.* **1**, 044007.

[bb97] Patterson, B. D. (2014). *Crystallogr. Rev.* **20**, 242–294.

[bb64] Powell, H. R., Johnson, O. & Leslie, A. G. W. (2013). *Acta Cryst.* D**69**, 1195–1203. 10.1107/S0907444912048524 PMC3689522 23793145

[bb65] Qiu, H. & Caffrey, M. (2000). *Biomaterials*, **21**, 223–234. 10.1016/s0142-9612(99)00126-x 10646938

[bb66] Ravelli, R. B., Leiros, H. K., Pan, B., Caffrey, M. & McSweeney, S. (2003). *Structure*, **11**, 217–224. 10.1016/s0969-2126(03)00006-6 12575941

[bb67] Redecke, L. *et al.* (2013). *Science*, **339**, 227–230.

[bb68] Roessler, C. G., Kuczewski, A., Stearns, R., Ellson, R., Olechno, J., Orville, A. M., Allaire, M., Soares, A. S. & Heroux, A. (2013). *J. Synchrotron Rad.* **20**, 805–808. 10.1107/S0909049513020372 PMC3747951 23955046

[bb69] Sauter, N. K., Hattne, J., Grosse-Kunstleve, R. W. & Echols, N. (2013). *Acta Cryst.* D**69**, 1274–1282. 10.1107/S0907444913000863 PMC3689530 23793153

[bb70] Sawaya, M. R. *et al.* (2014). *Proc. Natl. Acad. Sci. USA*, **111**, 12769–12774.

[bb71] Schlichting, I. (2015). *Nature*, **517**, 26–27. 10.1038/nature14072 25470040

[bb72] Schlichting, I., Almo, S. C., Rapp, G., Wilson, K., Petratos, K., Lentfer, A., Wittinghofer, A., Kabsch, W., Pai, E. F., Petsko, G. A. & Goody, R. S. (1990). *Nature*, **345**, 309–315. 10.1038/345309a0 2111463

[bb73] Schlichting, I. & Miao, J. (2012). *Curr. Opin. Struct. Biol.* **22**, 613–626. 10.1016/j.sbi.2012.07.015 PMC3495068 22922042

[bb74] Schotte, F., Cho, H. S., Kaila, V. R., Kamikubo, H., Dashdorj, N., Henry, E. R., Graber, T. J., Henning, R., Wulff, M., Hummer, G., Kataoka, M. & Anfinrud, P. A. (2012). *Proc. Natl. Acad. Sci. USA*, **109**, 19256–19261. 10.1073/pnas.1210938109 PMC3511082 23132943

[bb75] Schotte, F., Lim, M., Jackson, T. A., Smirnov, A. V., Soman, J., Olson, J. S., Phillips, G. N. Jr, Wulff, M. & Anfinrud, P. A. (2003). *Science*, **300**, 1944–1947. 10.1126/science.1078797 12817148

[bb76] Sierra, R. G. *et al.* (2012). *Acta Cryst.* D**68**, 1584–1587.

[bb77] Soares, A. S., Engel, M. A., Stearns, R., Datwani, S., Olechno, J., Ellson, R., Skinner, J. M., Allaire, M., & Orville, A. M. (2011). *Biochemistry*, **50**, 4399–4401. 10.1021/bi200549x PMC3144476 21542590

[bb78] Solem, J. C. (1986). *J. Opt. Soc. Am. B*, **3**, 1551–1565.

[bb79] Son, S.-K., Chapman, H. N. & Santra, R. (2011). *Phys. Rev. Lett.* **107**, 218102. 10.1103/PhysRevLett.107.218102 22181929

[bb80] Spence, J. C., Kirian, R. A., Wang, X., Weierstall, U., Schmidt, K. E., White, T., Barty, A., Chapman, H. N., Marchesini, S. & Holton, J. (2011). *Opt. Express*, **19**, 2866–2873. 10.1364/OE.19.002866 21369108

[bb81] Spence, J. C., Weierstall, U. & Chapman, H. N. (2012). *Rep. Prog. Phys.* **75**, 102601. 10.1088/0034-4885/75/10/102601 22975810

[bb82] Stellato, F. *et al.* (2014). *IUCrJ*, **1**, 204–212. 10.1107/S2052252514010070 PMC4107920 25075341

[bb83] Stevenson, H. P. *et al.* (2014). *Proc. Natl. Acad. Sci. USA*, **111**, 8470–8475.

[bb84] Suga, M., Akita, F., Hirata, K., Ueno, G., Murakami, H., Nakajima, Y., Shimizu, T., Yamashita, K., Yamamoto, M., Ago, H. & Shen, J.-R. (2015). *Nature*, **517**, 99–103. 10.1038/nature13991 25470056

[bb85] Sugahara, M. *et al.* (2014). *Nat. Methods*, **12**, 61–63.

[bb86] Tenboer, J. *et al.* (2014). *Science*, **346**, 1242–1246. 10.1126/science.1259357 PMC4361027 25477465

[bb87] Umena, Y., Kawakami, K., Shen, J. R. & Kamiya, N. (2011). *Nature*, **473**, 55–60. 10.1038/nature09913 21499260

[bb88] Wampler, R. D., Kissick, D. J., Dehen, C. J., Gualtieri, E. J., Grey, J. L., Wang, H. F., Thompson, D. H., Cheng, J. X. & Simpson, G. J. (2008). *J. Am. Chem. Soc.* **130**, 14076–14077. 10.1021/ja805983b PMC6905431 18831587

[bb89] Weierstall, U. (2014). *Philos. Trans. R. Soc. London Ser. B*, **369**, 20130337. 10.1098/rstb.2013.0337 PMC4052872 24914163

[bb90] Weierstall, U. *et al.* (2014). *Nat. Commun.* **5**, 3309. 10.1038/ncomms4309 PMC4061911 24525480

[bb91] Weierstall, U., Spence, J. C. & Doak, R. B. (2012). *Rev. Sci. Instrum.* **83**, 035108. 10.1063/1.3693040 22462961

[bb92] White, T. A., Barty, A., Stellato, F., Holton, J. M., Kirian, R. A., Zatsepin, N. A. & Chapman, H. N. (2013). *Acta Cryst.* D**69**, 1231–1240. 10.1107/S0907444913013620 PMC3689526 23793149

[bb93] White, T. A., Kirian, R. A., Martin, A. V., Aquila, A., Nass, K., Barty, A. & Chapman, H. N. (2012). *J. Appl. Cryst.* **45**, 335–341.

[bb94] Yano, J., Kern, J., Irrgang, K. D., Latimer, M. J., Bergmann, U., Glatzel, P., Pushkar, Y., Biesiadka, J., Loll, B., Sauer, K., Messinger, J., Zouni, A. & Yachandra, V. K. (2005). *Proc. Natl. Acad. Sci. USA*, **102**, 12047–12052. 10.1073/pnas.0505207102 PMC1186027 16103362

[bb95] Zarrine-Afsar, A., Barends, T. R., Muller, C., Fuchs, M. R., Lomb, L., Schlichting, I. & Miller, R. J. (2012). *Acta Cryst.* D**68**, 321–323. 10.1107/S0907444911055296 22349234

[bb96] Zarrine-Afsar, A., Muller, C., Talbot, F. O. & Miller, R. J. (2011). *Anal. Chem.* **83**, 767–773. 10.1021/ac1021024 21174439

